# Krüppel-like Factor 6 Suppresses the Progression of Pancreatic Cancer by Upregulating Activating Transcription Factor 3

**DOI:** 10.3390/jcm12010200

**Published:** 2022-12-27

**Authors:** Qunli Xiong, Zhiwei Zhang, Yang Yang, Yongfeng Xu, Ying Zhou, Su Zhang, Jinlu Liu, Ying Zheng, Qing Zhu

**Affiliations:** 1 Abdominal Oncology Ward, Cancer Center, West China Hospital, Sichuan University, Chengdu 610041, China; 2 State Key Laboratory of Biotherapy and Cancer Center, Sichuan University, Chengdu 610041, China

**Keywords:** Krüppel-like factor 6, pancreatic cancer, prognosis, proliferation, metastasis, activating transcription factor 3

## Abstract

Background: As a member of the Krüppel-like factor (KLFs) family, Krüppel-like factor 6 (KLF6) plays a critical role in regulating key cellular functions. Presently, scholars have proved the important role of KLF6 in the tumorigenesis of certain cancers through a large number of experiments. However, gaps still remain in our knowledge of the role of KLF6 in pancreatic cancer (PAAD). Therefore, this paper mainly investigates the role of KLF6 in the progression of pancreatic cancer. Methods: The expression pattern of KLF6 in pancreatic cancer was explored in pancreatic cancer tissues and cell lines. Then, we investigated the prognostic value of KLF6 in pancreatic cancer by immunohistochemical assays. Next, Cell Counting Kit-8 (CCK8) and clone information assays were employed to explore the proliferation of PAAD affected by KLF6. The metastasis and epithelial-mesenchymal transition (EMT) abilities affected by KLF6 were identified through transwell invasion as well as migration assays and western blots. Finally, the TRRUST tool was used to analyze the potential targeted genes of KLF6. The results were verified by Quantificational Real-time Polymerase Chain Reaction (qRT-PCR), western blot and rescue assays. Results: KLF6 expresses lowly in pancreatic cancer compared to corresponding normal tissues and relates to poor survival times. Overexpression of KLF6 inhibits the proliferation, metastasis, and EMT progression in pancreatic cancer cells. Further studies suggest that KLF6 could upregulate ATF3 in PAAD. Conclusions: Our results suggest that KLF6 can be a useful factor in predicting the prognosis of PAAD patients and that it inhibits the progression of pancreatic cancer by upregulating activating transcription factor 3 (ATF3).

## 1. Introduction

The Krüppel-like factor 6 (KLF6) gene, a member of the Krüppel-like factors (KLFs) family and located on chromosome 10, directly interacts with the deoxyribonucleic acid (DNA) sequence by either a GC box promoter component or a CACCC motif in its response promoter region [1,2]. Previous studies have proposed that the KLF6 gene is involved in the regulation of diverse forms of cellular progression covering cellular differentiation and growth [3,4,5], immunological and inflammatory responses [6,7], and tissue damage and repair [2,8]. Genetic alterations and the aberrant expression of KLF6 are correlated with the formation and progression of cancer [9]. In 2001, Narla et al. found that KLF6 was frequently inactivated in sporadic prostate cancer [10]. Subsequently, increasing studies have revealed that KLF6 could work as a tumor suppressor gene. For example, KLF6 was found to be inactivated and/or expression-downregulated in lung cancer [11], colorectal cancer [12], liver cancer [13], and some other cancers [14,15]. Further studies have revealed that knockdown KLF6 had positive effects on tumor progression, while KLF6 overexpression resulted in decreased tumorigenicity [16,17,18]. Nevertheless, an oncogenic role of KLF6 was addressed in recent studies [2]. For example, KLF6 is highly expressed in acute myeloid leukaemia and can be regulated by the pro-oncogenic fusion protein RUNX1-ETO to promote the progression of acute myeloid leukaemia [19]. Another study found that KLF6 could protect liver cancer from apoptosis to accelerate its progression [20]. However, the role of KLF6 in pancreatic cancer has not been identified.

Pancreatic cancer is one of the main cancer-related causes of death worldwide [21]. Among causes of cancer-related death, it will rank second by 2030 in the United States [22]. Patients with pancreatic cancer are often given a poor prognosis because most of them are not diagnosed until they have reached advanced stages of the disease [23]. Thus, finding novel therapeutic targets and/or biomarkers to improve the clinical outcome of pancreatic cancer is important. In this study, the expression pattern and the prognostic value of KLF6 in pancreatic cancer were explored. The role of KLF6 in proliferation and metastasis, as well as its potential mechanism in pancreatic cancer, were investigated as well.

## 2. Materials and Methods

### 2.1. PAAD Clinical Samples

We collected 57 formalin-fixed, paraffin-embedded cancer specimens from pancreatic cancer patients who underwent surgical excision between 2014 and 2018 at West China Hospital, Sichuan University (Chengdu, China). Clinical characteristics including age, gender, TNM stage (according to the 2018 eighth edition of the National Comprehensive Cancer Network staging criteria), tumor grade, and preoperative CA199 and CEA values were all collected. In total, the patients in our sample comprised 36 males and 21 females, with a mean age of 60.2 years, ranging from 43 to 76 years old. Within the sample, 1 case was well differentiated, 24 cases were moderately differentiated, and 32 cases were poorly differentiated. 2 patients were classified as stage I, 52 were classified as stage II, 1 was classified as stage III, and 2 were classified as stage IV. Follow-up data containing survival time and performance status were collected until September 2019. The median overall survival time of these 57 patients was 14 months, ranging from a minimum of 1 month to a maximum of 44 months. In addition to this sample, we collected 7 paired frozen pancreatic cancer and normal tissues from West China Hospital, Sichuan University. Patients were diagnosed clinically and pathologically with pancreatic ductal adenocarcinoma. None of these patients had been treated with radiotherapy or/and chemotherapy before surgery. This study was approved by the Institutional Ethics Committee of West China Hospital, Sichuan University, with authorized informed consent secured from all patients.

### 2.2. Immunohistochemistry (IHC)

As reported before [24], fixed tissue samples were subjected to paraffin embedding, sectioning, deparaffinating, rehydrating, and antigen retrieval. Following this, sections were incubated with a 3% hydrogen peroxide solution for 10 min at room temperature. Slides were then incubated with a KLF6 polyclonal antibody (Abcepta, Suzhou, China) at a dilution of 1:150 overnight at 4 °C. The next day, goat anti-rabbit antibody (Jackson, New York, NY, USA) at a dilution of 1:250 was added to the slides and incubated for 40 min at room temperature. A SignalStain DAB Substrate Kit (Cell Signaling Technology, Danvers, MA, USA) was used to for colorimetric detection. The stained sections were analyzed using a microscope (Pannoramic MIDI). The staining intensity was classified into four categories: 0, no staining; 1, weak; 2, moderate; 3, strong. The staining proportion was scored as follows: 0 indicating 0–5%; 1 indicating 6–25%; 2 indicating 26–50%; 3 indicating > 50%. The results of the intensity times the proportion of each slide were calculated, and a product > 3 was considered a high KLF6 expression, while a product ≤ 3 was considered a low KLF6 expression.

### 2.3. Cell Culture

AsPc-1, BxPC-3, Capan-1, Capan-2, CFPAC-1, MIA PaCa-2, PANC-1, and hTERT-HPNE cell lines were obtained from the cell bank of the Shanghai Institute of Cells, Chinese Academy of Science (Shanghai, China). Cells were cultured in either a Dulbecco Modified Eagle Medium (HyClone, Logan, UT, USA), a 1640 medium (HyClone), or an IMDM medium (Gibco, Billings, MT, USA) supplemented with 10% fetal bovine serum (Excell), 100 U/mL penicillin G, and 100 mg/mL streptomycin (Beyotime, Shanghai, China) at 37 °C in a humidified 5% CO_2_ incubator.

### 2.4. RNA Extraction, Reverse Transcription, and Quantitative Polymerase Chain Reaction (qRT-PCR)

RNA was isolated from cell lines and frozen tissues using a trizol reagent (Invitrogen, Thermofisher, Waltham, MA, USA) according to the manufacturer’s instructions. Reverse transcription was performed using a PrimeScript™ RT Master Mix (TaKaRa, Shiga, Japan). NovoStart^®^ SYBR qPCR SuperMix Plus (Novoprotein, Suzhou, China) was applied to conduct real-time quantitative PCR. The amplification procedure was set as follows: An initial denaturation at 95 °C for 30 s, followed by 40 cycles at 95 °C for 5 s, 60 °C for 30 s, and 72 °C for 30 s. The related target gene expression was normalized against β-actin by the relative quantification (2^−ΔΔCt^) method. 

The β-actin primers used were as follows: 

Forward: 5′-CACCATTGGCAATGAGCGGTTC-3′

Reverse: 5′-AGGTCTTTGCGGATGTCCACGT-3′

The used primers of the long mRNA of KLF6 were as follows: 

Forward: 5′-CAAGGGAAATGGCGATGCCT-3′

Reverse: 5′-CTTTTCTCCTGTGTGCGTCC-3′

The ATF3 primers used were as follows: 

Forward: 5′-CGCTGGAATCAGTCACTGTCAG-3′

Reverse: 5′-CTTGTTTCGGCACTTTGCAGCTG-3′

### 2.5. Western Blotting

Cells were collected and digested in an RIPA buffer in the presence of a 1% protease inhibitor cocktail (Bimake, Houston, TX, USA). Proteins were separated by SDS–PAGE and then transferred to PVDF membranes (Millipore, Burlington, MA, USA). After blocking in 5% skimmed milk, the membranes were incubated with antibodies to KLF6 (Abcepta), β-actin (Zhongshan Golden Bridge, Beijing, China), Tublin (Zhongshan Golden Bridge), E-cadherin (ZENBIO, Chengdu, China), N-cadherin (ZENBIO), Vimentin (ZENBIO), MMP2 (Proteintech, Wuhan, China), and ATF3 (ZENBIO) at 4 °C overnight. The next day, blots were rinsed before the application of a secondary antibody at room temperature for 90 min. Immunocomplexes were detected by electrochemiluminescence reagent (Millipore, Burlington, MA, USA), with β-actin or Tublin as a loading control.

### 2.6. Plasmid Construction, Lentiviral Transduction, and Generation of Stable Cell Lines

To establish stable KLF6-overexpression cell lines, human cDNA encoding full-length KLF6 gene was obtained by PCR amplification. KLF6 cDNA were subcloned into pLVX-NEO-3xFlag vector kindly provided by Dr. Zhou Zhao at Sichuan University. For the generation of lentiviral particles, the lentiviral vector and packaging DNA were co-transfected into 293T cells for 48–72 h, and the media containing lentiviruses were harvested and transduced to target cells. SiRNA was purchased from the Youkangjianxing Biotechnology Company (Chengdu, China). 5 nmol siRNA (siNC or siATF3) was transfected into pancreatic cancer cells by lipofectamine 3000 agents (Thermo) according to instructions.

### 2.7. CCK8 Assay and Clone Formation Assay

Briefly, cells (2 × 10^3^/well) were seeded onto 96-well plates and incubated for 0, 2, 4, and 6 days. 10 microliters of CCK8 reagent (TargetMol) per well were added and incubated at 37 °C for 2 h. Absorbance was measured at 450 nm under a multifunctional enzyme marker. For clone formation assays, transfected cells (2 × 10^3^/well) were cultured in 12-well plates for 10–14 days. Cells were fixed by application of 4% formaldehyde in PBS and stained with crystal violet.

### 2.8. Migration and Invasion Assays

The migration and invasive capability of pancreatic cancer cells were determined using transwell cell culture chambers with an 8-μm pore size polycarbonate membrane (Corning). In detail, cells (6 × 10^4^/well) with different treatments in 200 μL of serum-free medium were seeded into the upper chamber, while 800 μL of complete medium containing 10% FBS was placed in the lower chamber as a chemoattractant. As for the invasion assay, the membrane was coated with Matrigel™ (Corning, Corning, NY, USA) diluted at a 1:15 ratio with medium. After incubation at 37 °C for 24 h, the filters were fixed in 4% paraformaldehyde for 15 min and stained with crystal violet for 20 min. The cells on the upper side of the filter were wiped off gently by a cotton swab. 

### 2.9. Databases

TRRUST v2 (https://www.grnpedia.org/trrust/, accessed on 1 May 2022), an online dataset exploring the regulating network between transcription factor and genes [25], was used to find the targets of KLF6. Additionally, the GEPIA2 database (http://gepia2.cancer-pku.cn, accessed on 1 May 2022), an online tool for exploring gene expression pattern and interactive analyses [26], was utilized to analyze the relationship between the expression of KLF6 and target gene in this study.

### 2.10. Statistical Analysis

GraphPad Prism 5 and SPSS Statistics 26 were used for statistical analysis. Each experiment was repeated at least three times. Data were represented as mean ± SD, and Student’s *t*-test was applied for calculating *p* values. For studies of clinicopathologic parameters and for IHC signal quantification, chi-square tests and Fisher’s exact tests were applied to detect statistically significant associations with KLF6 expression. The Kaplan–Meier method was used to draw survival curves and *p* values were calculated using a log-rank test. Cox proportional hazards models were applied to conduct univariate and multivariate survival analyses. * indicates *p* < 0.05, ** indicates *p* < 0.01, *** indicates *p* < 0.001.

## 3. Results

### 3.1. Evaluating the Expression Patterns and Prognostic Value of KLF6 in PAAD Patients and Pancreatic Cancer Cell Lines

**Figure 1:**
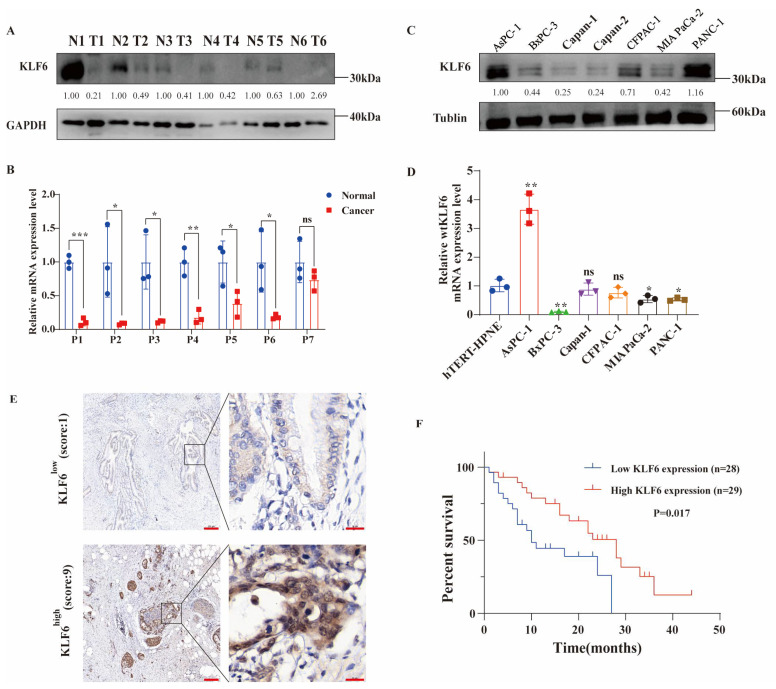
The expression pattern and prognostic value of KLF6 in pancreatic cancer. (**A**) KLF6 protein expression level comparison between tumor and paired normal tissues from 6 patients with pancreatic ductal adenocarcinoma. N: normal; T: tumor. (**B**) Relative KLF6 mRNA expression level comparison between tumor and paired normal tissues from 7 patients with pancreatic ductal adenocarcinoma. P: patient. Data represent mean ± SD; *n* = 3. * *p* < 0.05, ** *p* < 0.01, *** *p* < 0.001, ns means no significance. (**C**) KLF6 protein expression level in pancreatic cancer cell lines. (**D**) Relative mRNA expression of KLF6 in pancreatic cancer cell lines and a normal pancreatic ductal cell line. Data represent mean ± SD; *n* = 3. * *p* < 0.05, ** *p* < 0.01, ns means no significance. (**E**) Representative immunohistochemical staining images of low KLF6 expression and high KLF6 expression in pancreatic ductal adenocarcinoma tissues (original magnification of left pictures ×100; original magnification of right pictures ×1260; scale bar of left pictures 200 μm; scale bar of right pictures 20 μm). (**F**) Kaplan-Meier survival curves of patients with pancreatic ductal adenocarcinoma (*n* = 57).

Compared to paired normal tissues, tumor tissues express low protein and mRNA levels of KLF6 (Figure 1A,B). Additionally, protein levels of KLF6 are markedly different among pancreatic cancer cells (Figure 1C). Compared to an hTERT-HPNE cell, a normal pancreatic duct cell line, the mRNA of KLF6 is highly expressed in AsPc-1, while it is lowly expressed in BxPC-3, MIA PaCa-2, and PANC-1 (Figure 1D). To evaluate the prognostic potential for KLF6 expression in PAAD, we analyzed clinical specimens by IHC and found that the KLF6 immunostained signals in the PAAD tissue were markedly different among samples (Figure 1E). Furthermore, KM analysis revealed that PAAD patients with lower expression levels for KLF6 are positively associated with poor overall survival (Figure 1F). 

Next, we sought to analyze KLF6 expression profiles and clinicopathologic features in an independent collection of pancreatic cancer samples. As shown in Table 1, of 57 PAAD specimens, 28 (49.1 %) were considered high KLF6 expression, and 29 (50.9 %) were considered low KLF6 expression. Statistical analysis showed that KLF6 expression is independent of age (*p* = 0.896), sex (*p* = 0.470), TNM stage (*p* = 0.611), tumor grade (*p* = 0.223), preoperative CA199 value (*p* = 0.525), and preoperative CEA value (*p* = 0.631). In Table 2, univariate analysis with the Cox proportional hazards model identified low KLF6 expression (*p* = 0.017) and tumor grade with poor differentiation (*p* = 0.020) as statistically significant risk factors influencing the clinical survival of PAAD patients. Finally, we performed a multivariate analysis including TNM stage, which is considered an essential factor related to patient survival clinically, and discovered that both KLF6 expression (HR = 2.254, *p* = 0.033) and tumor grade (HR = 2.313, *p* = 0.026) were informative as prognostic markers for clinical outcome in patients (Table 3), indicating that KLF6 may be a potential prognosticator of PAAD prognosis. 

### 3.2. KLF6 Overexpression Inhibits Proliferation of Pancreatic Cancer Cells

**Figure 2:**
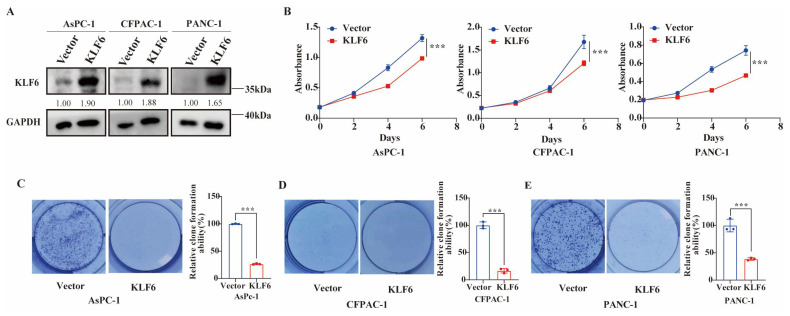
Overexpression of KLF6 inhibits proliferation of pancreatic cancer cells. (**A**) Western blot test results showing KLF6 protein expression level (KLF6-3xFlag) after KLF6-overexpression plasmid transfection of AsPc-1, CFPAC-1, and PANC-1 cells. (**B**) Cell proliferation of AsPc-1, CFPAC-1, and PANC-1 cells after KLF6 overexpression measured by CCK8 assays. Data represent mean ± SD; *n* = 6. *** *p* < 0.001. (**C**–**E**) Cell proliferation of AsPc-1, CFPAC-1, and PANC-1 cells after KLF6 overexpression measured by clone formation assays. Data represent mean ± SD; *n* = 3. *** *p* < 0.001.

Owing to the different expression of KLF6 between pancreatic normal cells and cancer cells, we further explored the potential function of KLF6 in PAAD. Overexpression KLF6 in AsPc-1, CFPAC-1, and PANC1 cell lines was performed. The results of the western blot test, as shown in Figure 2A, revealed that the protein level of KLF6 is overexpressed successfully. Then, CCK8 and clone formation assays were conducted to investigate the role of KLF6 in PAAD cell proliferation. As a result, overexpression of KLF6 in AsPc-1, CFPAC-1, and PANC1 cells decreases the growth of pancreatic cancer cells (Figure 2B–E).

### 3.3. KLF6 Overexpression Inhibits Metastasis of Pancreatic Cancer Cells

**Figure 3:**
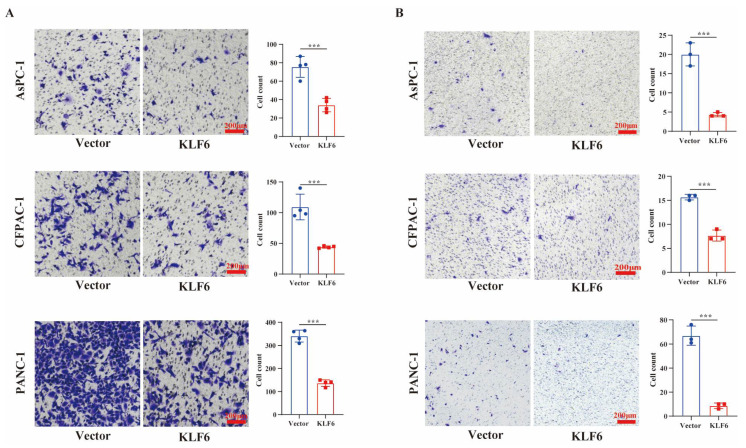
Overexpression of KLF6 inhibits metastasis of pancreatic cancer cells. (**A**) Cell migration ability of AsPc-1, CFPAC-1, and PANC-1 cells after KLF6 overexpression, respectively. Data represent mean ± SD; *n* = 4. *** *p* < 0.001. (**B**) Cell invasion ability of AsPc-1, CFPAC-1, and PANC-1 cells after KLF6 overexpression, respectively. Data represent mean ± SD; *n* = 3. *** *p* < 0.001.

To evaluate the effect of KLF6 on cell metastasis capability, we performed transwell migration and invasion assays. The number of AsPc-1, CFPAC-1, and PANC1 cells invading the lower side of the membrane decreased dramatically in those cells transfected with KLF6-overexpression plasmid compared to wild-type cells (Figure 3A). As for invasion ability, KLF6 overexpression markedly decreased the invasive ability of AsPc-1, CFPAC-1, and PANC1 cells as well (Figure 3B). These data suggest that KLF6 could effectively inhibit the malignant behaviors of pancreatic cancer cells.

### 3.4. Overexpression of KLF6 Impairs EMT Progression and Works by Upregulating ATF3 in Pancreatic Cancer Cell Lines

**Figure 4:**
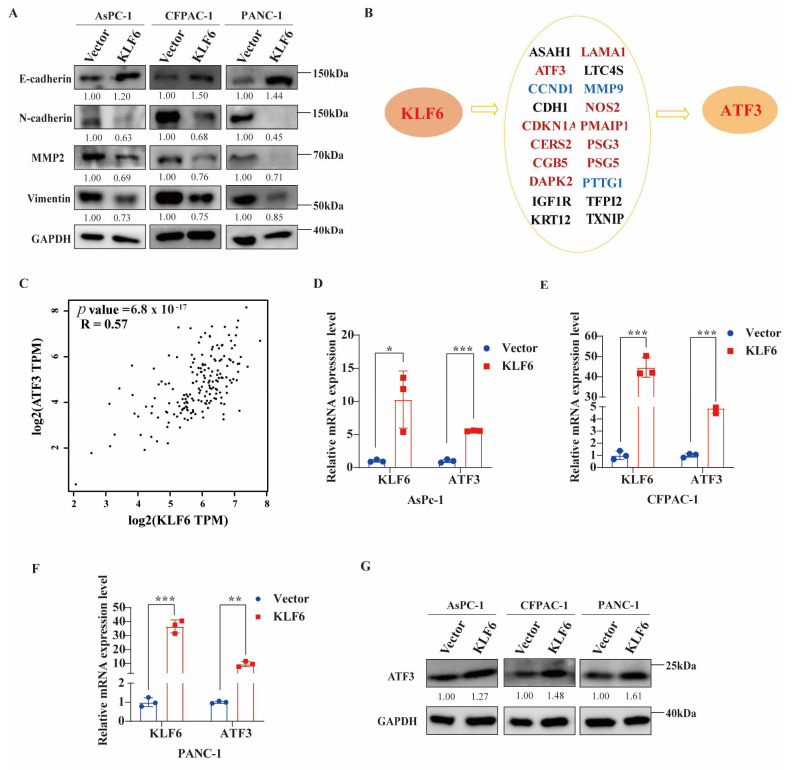
The EMT progression affected by overexpression of KLF6 and potential target genes of KLF6 in pancreatic cancer cells. (**A**) Western blot test results showing EMT protein markers, including E-cadherin, N-cadherin, MMP2, and vimentin, affected by overexpression of KLF6 in AsPc-1, CFPAC-1, and PANC-1 cells. (**B**) Potential targeted genes of KLF6 predicted by TRRUST database. Font in red refers to genes upregulated by KLF6; font in blue refers to genes downregulated by KLF6. (**C**) The correlation between KLF6 and ATF3 by GEPIA2 tool. (**D**–**F**) The mRNA ATF expression levels of ATF3 after overexpression of KLF6. Data represent mean ± SD; *n* = 3. * *p* < 0.05, ** *p* < 0.01, *** *p* < 0.001. (**G**) The protein ATF expression levels of ATF3 after overexpression of KLF6.

Next, the expressions of EMT marker proteins including E-cadherin (epithelial), N-cadherin, MMP2, and vimentin (mesenchymal) were investigated by western blot assays. The results demonstrated that the overexpression of KLF6 increased E-cadherin expression, simultaneously decreasing N-cadherin, MMP2, and vimentin expression (Figure 4A). The data above suggest that KLF6 overexpression reduced EMT progression in PAAD. To explore the potential mechanism of KLF6 in pancreatic cancer, the TRRUST database was used to determine possible targets of KLF6 and the regulatory relationship of KLF6 among them (Figure 4B). We determined that KLF6 can activate ATF3 (Activating Transcription Factor 3), CKDN1A (Cyclin Dependent Kinase Inhibitor 1A), CERS2 (Ceramide Synthase 2), CGB5 (Chorionic Gonadotropin Subunit Beta 5), DAPK2 (Death Associated Protein Kinase 2), LAMA1 (Laminin Subunit Alpha 1), NOS2 (Nitric Oxide Synthase 2), PMAIP1 (Phorbol-12-Myristate-13-Acetate-Induced Protein 1), PSG3 (Pregnancy Specific Beta-1-Glycoprotein 3), and PSG5 (Pregnancy Specific Beta-1-Glycoprotein 5), while repressing CCND1 (Cyclin D1), MMP9 (Matrix Metallopeptidase 9), and PTTG1 (PTTG1 Regulator of Sister Chromatid Separation, Securin). All of these were experimentally validated. ASAH1 (N-Acylsphingosine Amidohydrolase 1), CDH1 (Cadherin 1), IGF1R (Insulin-like Growth Factor 1 Receptor), KRT12 (Keratin 12), LTC4S (Leukotriene C4 Synthase), TFPI2 (Tissue Factor Pathway Inhibitor 2), and TXNIP (Thioredoxin Interacting Protein) are predicted to be regulated by KLF6. We have previously reported that decreased ATF3 expression is correlated with poor differentiation and shorter overall survival in PAAD patients, indicating that ATF3 is essential to the progression of pancreatic cancer (14). Therefore, we selected ATF3 for further analysis. Using the GEPIA2 tool, we found that the expression of KLF6 is positively associated with ATF3 (R = 0.57, *p* = 6.8 × 10^−17^: Figure 4C). Moreover, after KLF6 overexpression in AsPc-1, CFPAC-1, and PANC1 cells, the mRNA and protein expression levels of ATF3 are elevated (Figure 4D–G), suggesting that KLF6 may affect the progression of PAAD by upregulating ATF3.

**Figure 5:**
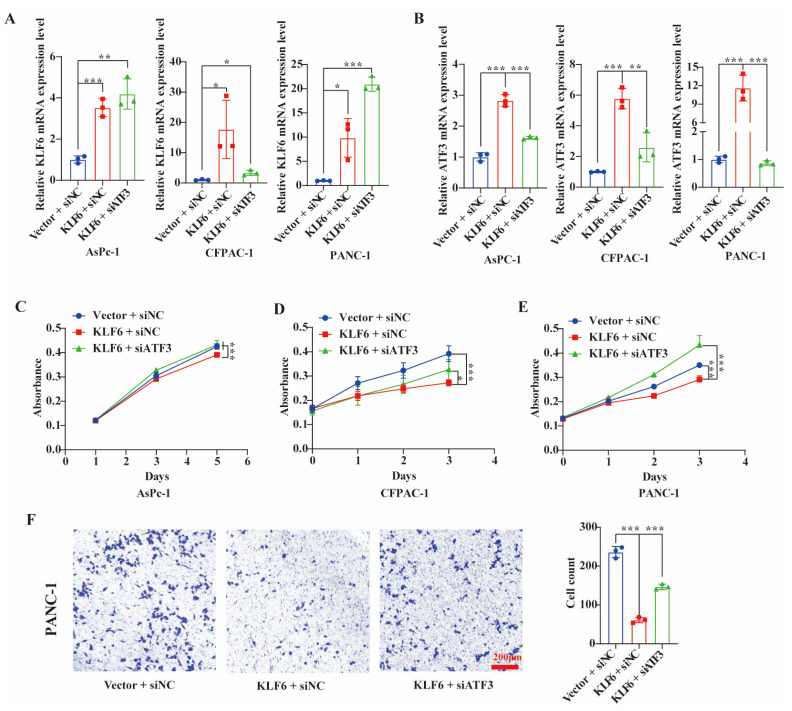
KLF6 inhibits the progression of pancreatic cancer through upregulating ATF3. (**A**) The relative mRNA expression levels of KLF6 after KLF6 overexpression or knockdown ATF3 in pancreatic cancer cells. Data represent mean ± SD; *n* = 3. * *p* < 0.05, ** *p* < 0.01, *** *p* < 0.001. (**B**) The relative mRNA expression levels of ATF3 after KLF6 overexpression or knockdown ATF3 in pancreatic cancer cells. Data represent mean ± SD; *n* = 3. ** *p* < 0.01, *** *p* < 0.001. (**C**–**E**) CCK8 assays were performed to measure the proliferation capacity after KLF6 overexpression or knockdown ATF3 in AsPC-1, CFPAC-1, and PANC-1. Data represent mean ± SD; *n* = 6. * *p* < 0.05, *** *p* < 0.001. (**F**) The migration capacity was measured after KLF6 overexpression or knockdown ATF3 in AsPC-1, CFPAC-1, and PANC-1. Data represent mean ± SD; *n* = 3. *** *p* < 0.001.

To determine whether KLF6 is involved in pancreatic cancer progression through ATF3, we performed rescue experiments (Figure 5A,B). The CCK8 results showed that silencing ATF3 could partially or completely rescue the decreased cell proliferation induced by KLF6 overexpression in AsPc-1, CFPAC-1, and PANC-1 cells (Figure 5C–E). The migration result showed that knockdown of ATF3 partially rescued the decreased cell migration induced by KLF6 overexpression in PANC-1 cells (Figure 5F). These results suggest that KLF6 is involved in the progression of pancreatic cancer through upregulating ATF3.

## 4. Discussion

The human KLF genes, encoding Krüppel-like factors with zinc finger DNA-binding proteins, have been reported to play a vital role in cellular processes [1,2,9]. Playing a central role in modulating these processes, the human *KLF6* gene has been reported to influence tumorigenesis and the development of cancer [9]. Previously, several studies identified *KLF6* as a tumor-suppressor gene due to frequent somatic inactivation of the *KLF6* gene and/or downregulation of KLF6 expression in prostate carcinoma, glioblastoma, colorectal tumors, gastric cancer, hepatocellular carcinoma, and lung cancer [10,11,17,27,28,29]. In contrast to these studies, several groups proposed that genetic alterations of KLF6 were infrequently observed and overexpressed in some types of cancer [30,31,32,33,34]. In this research, we found that KLF6 expresses lowly in pancreatic cancer samples and that survival analysis demonstrates that lower KLF6 expression is linked to a worse survival prognosis in PAAD. This indicates that KLF6 is a promising molecular biomarker for clinical survival prognosis. We also verified that KLF6 inhibits growth and metastasis in pancreatic cancer, indicating that KLF6 plays a tumor-suppressor role in pancreatic cancer. It should be noted that the KLF6 analyzed in our research was the longest isoform. Studies have reported that a splice variant of KLF6, KLF6-SV1, is a cancer-promoting gene in ovarian and prostate cancer models [15,35]. In 2008, Hartel et al. reported that enhanced alternative splicing of the KLF6 gene positively correlates with better prognosis in patients with pancreatic cancer samples, and the increased alternative splicing of KLF6 was primarily due to enhanced splice form expression rather than reduced KLF6 full length mRNA, indicating alternative splicing of KLF6 as a growth-promoting mechanism in human cancer. Our study identifies the tumor-suppressor role of KLF6 in pancreatic cancer, yet the role of splicing isoforms of KLF6 in pancreatic cancer needs further research.

KLF6, as a transcription factor, can interact with other proteins and regulate the transcription of its downstream targets to participate in the tumorigenesis development. Additionally, the mRNA of KLF6 receives the regulation of microRNA. For example, KLF6 can be targeted by miRNA-630 to regulate the growth and invasion in ovarian cancer [36]. Recent studies have illustrated the ability of KLF6 to recruit and interact with other transcription factors, such as p53 (Tumor Protein P53), RUNX1 (runt-related transcription factor 1), E2F1 (E2F Transcription Factor 1), and HDAC3 (histone deacetylase 3) [19,37,38,39]. Additionally, KLF6 could regulate the expression of ATF3, which is a member of the ATF/CREB family of transcription factors and which is involved in many cellular functions, including cell proliferation and metastasis [40]. For example, Huang et al. found that KLF6 can induce apoptosis in prostate cancer by modulating ATF3 [41]. Our results showed that knockdown ATF3 partially or completely rescues the reduced cell proliferation and migration induced by KLF6 overexpression, suggesting that KLF6 functions partly through ATF3. However, the specific and detailed molecular mechanisms of how ATF3 is regulated by KLF6 and other potential mechanisms in pancreatic cancer need more research. Meanwhile, these results also indicate the potential function of ATF3 on pancreatic cancer cell. In a word, this study demonstrates that KLF6 inhibits the progression of pancreatic cancer through upregulating ATF3 and may serve as a potential therapeutic target. Nevertheless, more preclinical studies are needed before this is possible.

## 5. Conclusions

Our work investigated the essential role of KLF6 in pancreatic cancer. The results indicate that KLF6 is lowly expressed in tumor samples when compared to normal samples, and its expression is closely correlated with clinical prognosis in pancreatic cancer. Furthermore, KLF6 can inhibit the progression of pancreatic cancer by upregulating ATF3. These findings may better clarify the potential value of KLF6 in tumorigenesis and progression, and provide a novel perspective for a more precise treatment of pancreatic cancer in the future.

## Figures and Tables

**Table 1:** Association of KLF6 expression and clinicopathologic parameters.

Parameters		*n*	Low Expression	High Expression	*p* Value
Number of patients		57	28	29	
Age	≤60	28	14	14	0.896
	>60	29	14	15	
Sex	Male	36	19	17	0.470
	Female	21	9	12	
TNM stage	I-II	54	26	28	0.611
	III-IV	3	2	1	
Grade	Well and moderate	25	10	15	0.223
	Poor	32	18	14	
Diabetes	Yes	11	5	6	0.786
	No	46	23	23	
Smoke	Yes	22	13	9	0.233
	No	35	15	20	
Drinking	Yes	17	12	5	0.035 *
	No	40	16	24	
Preoperative CA19-9 value	≥37	47	24	23	0.525
	<37	10	4	6	
Preoperative CEA value	≥5	18	8	10	0.631
	<5	39	20	19	

* *p* < 0.05 was considered statistically significant.

**Table 2:** Univariate Cox proportional hazards analysis for survival of PAAD patients.

Variable	Hazard Ratio	95% Confidence Interval	*p* Value
KLF6 expression (low/high)	2.431	1.166–5.070	0.017 *
Age (≤60/>60)	0.819	0.424–1.581	0.552
Gender (female/male)	0.605	0.296–1.236	0.168
TNM stage (I–II/III–IV)	0.503	0.150–1.689	0.266
Grade (poor/moderate and well)	2.316	1.138–4.711	0.020 *
Preoperative CA19-9 value (<37/≥37)	0.556	0.213–1.450	0.230
Preoperative CEA value (<5/≥5)	0.570	0.272–1.192	0.135

* *p* < 0.05 was considered statistically significant.

**Table 3:** Multivariate Cox proportional hazards analysis for survival of PAAD patients.

Variables	Hazard Ratio	95.0% CI	*p* Value
TNM stage	0.330	0.094–1.163	0.085
Grade	2.313	1.103–4.850	0.026 *
KLF6 expression	2.254	1.068–4.757	0.033 *

* *p* < 0.05 was considered statistically significant.

## Data Availability

Not applicable.
